# Assessment of dehydroepiandrosterone sulfate (DHEA-S) suppression following the 1 mg overnight dexamethasone suppression test in women with hirsutism and elevated DHEA-S

**DOI:** 10.20945/2359-4292-2026-0107

**Published:** 2026-09-11

**Authors:** Hamide Piskinpasa, Didem Acarer Bugun, Mehmet Emin Piskinpasa, Sema Ciftci, Ilkay Cakir

**Affiliations:** 1 Department of Endocrinology and Metabolism, Bakirkoy Dr. Sadi Konuk Training and Research Hospital, Istanbul, Turkey; 2 Department of Endocrinology and Metabolism, Basaksehir Cam and Sakura City Hospital, Istanbul, Turkey; 3 Department of Internal Medicine, Istanbul Training and Research Hospital, Istanbul, Turkey

**Keywords:** Hirsutism, DHEA-S, dexamethasone suppression test, adrenal androgen autonomy, functional adrenal hyperandroge­nism, PMOS

## Abstract

**Objective:**

The utility of the 1-mg overnight dexamethasone suppression test (DST) for assessing dehydroepiandrosterone sulfate (DHEA-S) suppressibility is not well defined. Hence, this study aimed to assess DHEA-S response and identify predictors of non-suppression in hirsute women with elevated DHEA-S levels.

**Subjects and methods:**

This retrospective study included 362 hirsute women with elevated DHEA-S. Suppression was defined as a post-DST DHEA-S level normalized to the age-specific upper limit of normal (ULN) or a decrease of 50% from baseline. Patients were classified as suppressed (Group 1) or non-suppressed (Group 2). Logistic regression and receiver operating characteristic curve analysis were used to identify predictors of suppression, including the “percent excess” (% excess) above the ULN.

**Results:**

DHEA-S was suppressed in 65.5% of patients (Group 1), while 34.5% was not (Group 2). The non-suppressed group had significantly higher levels of total testosterone, a greater frequency of total testosterone >ULN, and higher 17-hydroxyprogesterone (17-OHP) levels. No differences were observed in luteinizing hormone, follicle-stimulating hormone, or homeostatic model assessment of insulin resistance. Logistic regression confirmed that both % excess (odds ratio [OR] = 0.947) and DHEA-S levels >700 µg/dL (OR = 0.069) were inversely associated with suppression. Receiver operating characteristic analysis for % excess showed good predictive performance (area under the curve = 0.827), with an optimal cut-off of 32.22%.

**Conclusion:**

Hirsute women with elevated DHEA-S, 34.5% exhibited a lack of DHEA-S suppression on the 1-mg DST. The % excess (cut-off >32.22%) and an absolute DHEA-S threshold of >700 µg/dL may help identify this subgroup, which was associated with higher total testosterone and 17-OHP levels despite similar luteinizing hormone levels. Overall, the lack of DHEA-S suppression on the 1-mg DST, together with higher total testosterone and 17-OHP despite similar luteinizing hormone, suggests a possible functional adrenal contribution to the hyperandrogenic phenotype.

## INTRODUCTION

Hirsutism is a well-known clinical manifestation of hyperandrogenism that requires a thorough differential diagnosis to determine the underlying etiology (^1^). Hirsutism, characterized by the excessive growth of terminal hair in a male-pattern distribution (e.g., face, chest, back), is a common clinical condition that can significantly impair quality of life in women (^1^,^2^). In most cases, the underlying cause is hyperandrogenism, defined by the overproduction of androgens or increased tissue sensitivity to them (^1^,^2^). The most frequent cause of hyperandrogenism is polyendocrine metabolic ovarian syndrome (PMOS), which affects a substantial proportion of women of reproductive age (^1^,^3^).

The source of hyperandrogenism is typically ovarian or adrenal. While total testosterone reflects androgen production from the ovaries and adrenal glands, dehydroepiandrosterone sulfate (DHEA-S) is secreted almost exclusively by the adrenal glands (^2^,^4^). Therefore, serum DHEA-S is considered a specific biochemical marker of adrenal androgen production. In a patient presenting with hirsutism and hyperandrogenism, elevated DHEA-S levels suggest that the underlying pathology may be of adrenal origin (^1^,^3^,^5^). The differential diagnosis for elevated DHEA-S includes PMOS (with an adrenal component), late-onset congenital adrenal hyperplasia (LOCAH), Cushing’s syndrome (CS), or, rarely, androgen-secreting adrenal tumors (^1^,^3^). Because the clinical management and prognosis of these conditions differ significantly, accurate identification of the etiology is critical (^1^,^3^).

The 1 mg overnight dexamethasone suppression test (DST) is a standard diagnostic tool widely used to evaluate the hypothalamic-pituitary-adrenal axis, primarily for screening for CS (^5^). The purpose of this test is to determine whether adequate suppression of serum cortisol occurs following dexamethasone administration, which inhibits the release of adrenocorticotropic hormone (ACTH) from the pituitary gland (^3^,^5^). DHEA-S is also secreted by the adrenal cortex under ACTH regulation (^3^,^5^). However, the role and clinical utility of the 1 mg DST in assessing suppression of adrenal androgens, particularly DHEA-S, are not as clear as for cortisol evaluation. Although CS is a rare cause of hirsutism, the 1-mg overnight DST may be performed in selected patients when clinical features or biochemical findings raise suspicion for hypercortisolism (^5^).

In our clinical practice, when the 1-mg DST was performed as part of the evaluation of women with hirsutism, post-dexamethasone DHEA-S levels were also assessed in those with elevated baseline DHEA-S. Limited data exist on whether changes in elevated DHEA-S levels after DST could serve as a diagnostic tool for distinguishing adrenal-origin hyperandrogenism (^6^–^8^). Nevertheless, given the prolonged serum half-life of DHEA-S (~10–16 h) and reports that longer dexamethasone regimens may be required to reliably suppress DHEA-S, the discriminatory performance of a 1 mg overnight DST protocol for DHEA-S must be interpreted with caution. Some studies suggest that even among patients who achieve adequate cortisol suppression, DHEA-S responses may vary, possibly indicating partial or selective adrenal androgen autonomy (^6^–^8^).

Given the above, we aimed to evaluate the DHEA-S response to the 1 mg overnight DST in women with hirsutism and baseline DHEA-S levels above age-specific reference ranges. Because DHEA-S levels decline with age at similar rates in both women with PMOS and healthy controls, age-adjusted analyses were performed to identify predictors of DHEA-S suppression using the percent excess (% excess) of baseline DHEA-S above the age-specific upper limit of normal (ULN).

## SUBJECTS AND METHODS

This retrospective study was conducted in accordance with the Declaration of Helsinki and consisted of reviewing the medical records of women with hirsutism and serum DHEA-S levels exceeding age-specific reference ranges between January 2018 and December 2024. A total of 362 patients who underwent the 1-mg overnight DST were included.

Exclusion criteria included age <18 years; pregnancy or postmenopausal status; current use of corticosteroids, oral contraceptives, or antiandrogenic medications; known adrenal or ovarian tumors or prior diagnosis of CS; presence of other endocrinopathies (e.g., hyperprolactinemia, hypothyroidism); chronic systemic illnesses (e.g., hepatic or renal failure, malignancy); incomplete hormonal data; and failure to suppress cortisol below 1.8 µg/dL after the 1 mg overnight DST.

All patients underwent comprehensive evaluation by experienced endocrinologists and gynecologists. Hirsutism was defined by a Ferriman-Gallwey score ≥9 (^3^). All patients had a confirmed diagnosis of hirsutism and available serum DHEA-S measured at both baseline and after the 1 mg DST. Blood samples were collected during the early follicular phase of the menstrual cycle (days 2–5) following an overnight fast. The baseline hormonal panel included fasting glucose and insulin, thyroid-stimulating hormone (TSH), luteinizing hormone (LH) and follicle-stimulating hormone (FSH), estradiol and prolactin, total testosterone and 17-hydroxyprogesterone (17-OHP), and DHEA-S.

All DHEA-S measurements were performed in the same institutional laboratory using a single immunoassay platform (Access DHEA-S, Beckman Coulter, Inc.). Age-specific ULNs were defined according to the 95^th^ percentile reference intervals provided in the manufacturer’s instructions for use. For premenopausal women, the ULN values used were: 321 µg/dL (ages 18–20), 391 µg/dL (ages 21–30), 266 µg/dL (ages 31–40), and 231 µg/dL (ages 41–50).

To exclude CS as part of the differential diagnosis of hirsutism, all patients underwent the 1 mg overnight DST. Since elevated DHEA-S levels were also present in our study population, the 1 mg overnight DST was also utilized to assess adrenal androgen suppression. Patients were informed to take 1 mg of oral dexamethasone at 11:00 pm, and blood samples for serum cortisol and DHEA-S were collected the following morning between 8:00 and 9:00 am after an overnight fast. Following the test, serum cortisol and DHEA-S levels were recorded. Adequate suppression was defined as a morning serum cortisol level <1.8 µg/dL (50 nmol/L).

Patients with a baseline 17-OHP level above 2 ng/mL underwent a 250 µg ACTH stimulation test to rule out LOCAH. In patients with a baseline DHEA-S level exceeding 700 µg/dL, non-contrast adrenal magnetic resonance imaging was performed to rule out adrenal masses. Menstrual irregularity was defined according to the 2023 guidelines as fewer than eight menstrual cycles per year or cycle lengths (<21 or >35 days) (^3^). PMOS was diagnosed according to the same 2023 evidence-based guidelines, requiring at least two of the following three features after exclusion of other etiologies: (i) oligo/anovulation, (ii) clinical and/or biochemical hyperandrogenism, and (iii) polycystic ovarian morphology (PCOM) on ultrasound (^3^); PCOM was defined as ≥20 follicles per ovary or an ovarian volume >10 mL (^3^).

Homeostatic model assessment of insulin resistance (HOMA-IR) was calculated as [glucose (mg/dL) × insulin (µU/mL)] / 405. The LH/FSH ratio was also recorded. A HOMA-IR value above ~2.5 was considered consistent with insulin resistance. The % excess of baseline DHEA-S above the age-specific ULN (%) and the % change in DHEA-S after the 1 mg DST (%) were calculated using Equations 1 and 2, respectively:


(1) % excess=[(baseline DHEA-S - age-specific ULN for DHEA-S)/(age-specific ULN for DHEA-S)]×100

(2) % change=[(baseline DHEA-S - DHEA-S levels after 1 mg DST)/(baseline DHEA-S)]×100

Patients were classified into two groups based on the DHEA-S response to the DST; Group 1 (DHEA-S suppressed) comprised patients whose post-DST DHEA-S level either normalized to the age-specific ULN or decreased by 50% from baseline and Group 2 (DHEA-S non-suppressed) comprised patients who did not meet the criteria for suppression. Group comparisons were made based on demographic, clinical, and biochemical parameters. Logistic regression analysis was conducted to evaluate variables potentially associated with DHEA-S suppression following the 1 mg DST.

### Statistical analysis

Statistical analyses were performed using IBM SPSS Statistics (v. 27). Categorical variables were presented as frequencies and percentages; continuous variables as mean ± standard deviation. The Kolmogorov-Smirnov test was used to assess the normality of distribution. For independent-group comparisons, Student’s t-test was applied to normally distributed variables, and the Mann-Whitney U test to non-normally distributed variables. Categorical variables were compared using the chi-square test or Fisher’s exact test, as appropriate. Bivariate correlations were assessed using Pearson’s correlation for normally distributed variables and Spearman’s rank correlation otherwise. Binary logistic regression analysis was performed to evaluate variables potentially associated with DHEA-S suppression after the 1 mg DST. Results were presented as odds ratios (ORs) with 95% confidence intervals (CIs). Receiver operating characteristic (ROC) curve analysis was performed to assess the ability of baseline DHEA-S, expressed as % excess over the age-specific ULN, to predict suppression after the 1-mg DST. The optimal cut-off value was determined according to the Youden index. Statistical significance was defined as *p* < 0.05.

## RESULTS

A total of 362 female patients with hirsutism and elevated baseline serum DHEA-S were included in the study. The mean age of the study cohort was 24.44 ± 6.30 years, and the mean baseline DHEA-S level was 497.16 ± 119.58 µg/dL. Following administration of the 1-mg overnight DST, DHEA-S levels decreased in all participants. The mean post-DST DHEA-S level was 340.13 ± 91.93 µg/dL, corresponding to a mean absolute reduction of 157.03 µg/dL and a mean % change of 30.67 ± 15.34%. After the DST, DHEA-S levels normalized in 235 patients (64.9%). Thirty-one patients (8.6%) showed a ≥50% reduction from baseline; however, 29 of these patients were also among those who achieved normalization. Thus, only two additional patients met the suppression criterion based solely on a ≥50% reduction, resulting in a total of 237 patients (65.5%) classified as having DHEA-S suppression.

Based on predefined criteria, 237 patients (65.5%) were classified as Group 1 (DHEA-S suppressed), and 125 patients (34.5%) as Group 2 (DHEA-S non-suppressed). As expected, Group 2 exhibited significantly higher baseline DHEA-S levels, higher % excess of baseline DHEA-S above the ULN, and higher post-DST DHEA-S levels compared to Group 1 (*p* < 0.001). Conversely, the % change in DHEA-S was significantly greater in Group 1 (*p* < 0.001). Total testosterone and 17-OHP levels were statistically higher in Group 2 than in Group 1 (*p* = 0.006 and *p* = 0.039, respectively). Elevated total testosterone (>ULN) was more frequently observed in Group 2 (*p* = 0.026). The two groups did not differ significantly in age, glucose, insulin, HOMA-IR, TSH, LH, FSH, estradiol, or prolactin. Detailed demographic and hormonal comparisons between groups are presented in Table 1.

**Table 1.** Comparison of clinical and laboratory findings between patients with and without DHEA-S suppression (n = 362) after the 1-mg overnight dexamethasone suppression test

Variable	DHEA-S suppression (n = 237)	DHEA-S non-suppression (n = 125)	*p*-Value
Age, years	24.37 ± 5.98	24.58 ± 6.88	ns
PCOM on ultrasonography, n (%)	136 (57)	70 (56)	ns
Glucose, mg/dL	89.99 ± 8.58	90.41 ± 10.29	ns
Insulin, µU/mL	12.85 ± 12.57	12.63 ± 10.61	ns
HOMA-IR*	5.20 ± 3.47	4.44 ± 2.09	ns
TSH, mIU/L	2.21 ± 1.39	2.10 ± 1.14	ns
LH, IU/L	7.08 ± 3.87	6.57 ± 3.51	ns
FSH, IU/L	6.06 ± 1.56	6.12 ± 1.82	ns
LH/FSH ratio	1.24 ± 0.75	1.25 ± 1.02	ns
Estradiol, pg/mL	44.37 ± 36.64	44.85 ± 31.21	ns
Prolactin, ng/mL	20.07 ± 12.91	20.24 ± 10.95	ns
Total testosterone, ng/mL	0.60 ± 0.24	0.68 ± 0.27	0.006^‡^
Total testosterone above the age-specific ULN, n (%)	6 (2)	10 (8)	0.026^‡^
17-OHP, ng/mL	1.84 ± 1.46	2.12 ± 1.30	0.039^‡^
DHEA-S (baseline), µg/dL	465.18 ± 107.37	557.80 ± 118.43	<0.001^‡^
% excess DHEA-S (baseline) above the age-specific ULN, %**	27.50 ± 28.21	57.57 ± 29.84	<0.001^‡^
DHEA-S >700 µg/dL, n (%)	9 (4)	15 (12)	0.006^‡^
DHEA-S (after 1-mg DST), µg/dL	292.78 ± 57.84	429.55 ± 76.60	<0.001^‡^
Cortisol (after 1-mg DST), µg/dL	0.67 ± 0.27	0.67 ± 0.23	ns
Percent change in DHEA-S after the 1-mg DST (%)***	35.52 ± 14.67	21.48 ± 12.08	<0.001^‡^

Data are given as mean ± standard deviation.

‡ Statistically significant (*p* < 0.05).

* HOMA-IR: [(glucose × insulin) / 405];

** % excess: [(baseline DHEA-S - age-specific ULN for DHEA-S)/(age-specific ULN for DHEA-S)] × 100;

*** percent change: [(baseline DHEA-S - DHEA-S levels after 1 mg DST)/(baseline DHEA-S)] × 100. ns: No statistical significance; DHEA-S: dehydroepiandrosterone sulfate; DST: dexamethasone suppression test; FSH: follicle stimulating hormone; HOMA-IR: homeostatic model assessment of insulin resistance; LH: luteinizing hormone; TSH: thyroid stimulating hormone; ULN: upper limit of normal; PCOM: polycystic ovary morphology; 17-OHP: 17-hydroxyprogesterone.

Twenty-four patients (6.6%) had baseline DHEA-S levels of >700 µg/dL. In this specific subgroup (n = 24), the mean baseline DHEA-S was 802.17 ± 99.53 µg/dL. Following the 1-mg DST, the mean post-DST DHEA-S remained high at 443.33 ± 123.74 µg/dL, corresponding to a mean % change of 43.39% ± 19.07%. Within this subgroup, six patients (25.0%) achieved normalization of DHEA-S to the age-specific ULN, and an additional three patients (12.5%) had a reduction of 50% from baseline. Magnetic resonance imaging was performed in 20 patients; no adrenal masses or hyperplasia were identified. Among patients with baseline 17-OHP levels above 2 ng/mL (n = 45), an ACTH stimulation test was performed; two patients (4.4%) were diagnosed with LOCAH. Both were classified in the DHEA-S suppressed group after the DST. The first patient had a baseline DHEA-S of 525 µg/dL, which decreased to 257 µg/dL after DST; the second had a baseline DHEA-S of 413 µg/dL, decreasing to 341 µg/dL after DST.

Menstrual irregularities were observed in 37% of the patients (n = 134), and PCOM was present in 57% (n = 206). PMOS was diagnosed in 79% (n = 287) of patients. In non-parametric correlation analysis assessing sources of hyperandrogenism, the % excess of baseline DHEA-S showed statistically significant, positive, and weak associations with total testosterone (r = 0.221, *p* < 0.001) and 17-OHP (r = 0.133, *p* = 0.025). Multivariable binary logistic regression identified factors associated with DHEA-S suppression after the 1-mg DST (i.e., Group 1 assignment). Both the % excess of baseline DHEA-S above the age-specific ULN and DHEA-S >700 µg/dL were inversely associated with suppression (OR = 0.947, 95% CI: 0.923–0.972, *p* < 0.001; OR = 0.069, 95% CI: 0.013–0.352, *p* = 0.001, respectively). Patients with DHEA-S >700 µg/dL were 14.5 times more likely to exhibit non-suppression. Age, total testosterone, 17-OHP, and baseline DHEA-S were not significantly associated with suppression (*p* > 0.05). Full regression analysis results are provided in Table 2.

**Table 2.** Logistic regression analysis of variables associated with DHEA-S suppression after 1-mg dexamethasone suppression test

Variables	B	SE	OR	*p*-Value	95% CI	
					Lower	Upper
Age, years	0.043	0.033	1.043	0.196	0.978	1.113
17-OHP, ng/mL	0.060	0.117	1.061	0.611	0.843	1.336
Total testosterone, ng/mL	-0.970	0.674	0.379	0.150	0.101	1.419
DHEA-S (baseline), µg/dL	0.004	0.003	1.004	0.257	0.997	1.010
% excess DHEA-S (baseline) above the age-specific ULN, %*	-0.055	0.013	0.947	<0.001^‡^	0.923	0.972
Baseline DHEA-S >700 µg/dL	-2.678	0.834	0.069	0.001^‡^	0.013	0.352

‡ Statistically significant (*p* < 0.05).

* % excess: [(baseline DHEA-S - age-specific ULN for DHEA-S)/(age-specific ULN for DHEA-S)] × 100. CI: Confidence interval; DHEA-S: dehydroepiandrosterone sulfate; OR: odds ratio; SE: standard error; ULN: upper limit of normal; 17-OHP: 17-hydroxyprogesterone

**Figure 1.**
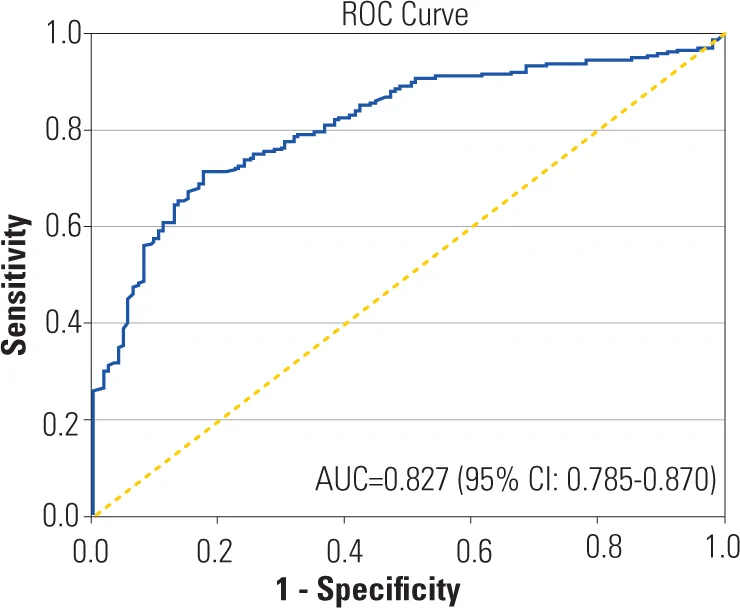
ROC curve demonstrating the diagnostic performance of basal DHEA-S expressed as percent excess over the age-specific upper limit of normal in predicting DHEA-S suppression after the 1-mg overnight dexamethasone suppression test.

ROC curve analysis (Figure 1) of the % excess of baseline DHEA-S to predict suppression showed good discriminative performance, with an area under the curve of 0.827 (95% CI: 0.785–0.870, *p* < 0.001). The optimal cut-off for % excess of baseline DHEA-S was 32.22%, yielding a sensitivity of 72.3% and specificity of 82.7% for predicting suppression.

## DISCUSSION

This study evaluated the DHEA-S response to a 1-mg overnight DST in 362 hirsute women with elevated baseline DHEA-S. Our main finding is that adrenal androgen suppressibility to dexamethasone is heterogeneous: while most participants (65.5%) showed adequate suppression (50% decrease and/or normalization), a substantial subgroup (34.5%) did not. Notably, this non-suppressed subgroup exhibited higher total testosterone and 17-OHP levels despite similar LH, suggesting a distinct biochemical phenotype with a possible functional adrenal contribution to hyperandrogenism.

The 1-mg DST is central to screening for CS by assessing cortisol suppressibility (^5^). Its role in evaluating adrenal androgen dynamics, however, is less established (^5^–^9^). Approximately 50% of women with PMOS demonstrate an exaggerated adrenal androgen response to exogenous ACTH administration, suggesting increased adrenal sensitivity to ACTH stimulation (^10^). The clinical presentation of CS varies widely due to differences in cortisol excess and glucocorticoid receptor sensitivity. Menstrual disturbances affect up to 80% of women with CS, nearly half of whom exhibit PMOS features (^9^). Historically, the 48-hour low-dose DST (LDDST) has been used to differentiate causes of androgen excess (^6^,^8^). In one study of 51 women with PMOS who underwent LDDST, 27.4% (14/51) had elevated baseline DHEA-S, yet both cortisol and DHEA-S were markedly suppressed to normal ranges after testing (^6^). Another study including 211 women with nontumorous hyperandrogenism and 17 patients with histologically confirmed androgen-secreting tumors reported elevated DHEA-S in 69% of tumor cases and 47% of nontumorous cases (^8^). Following LDDST, DHEA-S levels were adequately suppressed (≥40% reduction or normalization) in 92% of patients with nontumorous hyperandrogenism and two patients with ovarian tumors (^8^). LDDST thus achieved 100% sensitivity and 88% specificity in identifying androgen-secreting tumors (^8^), comparable to earlier data from longer dexamethasone protocols (3 mg/day for 5 days), in which DHEA-S suppression was complete in all patients with benign hyperandrogenism but absent in those with adrenal carcinoma (^7^).

A key methodological choice in our study was to use the 1-mg overnight DST rather than the multi-day LDDST. Unlike longer dexamethasone protocols, the 1-mg overnight DST is primarily used to assess cortisol suppressibility in selected patients with suspected hypercortisolism. In our clinical practice, when the 1-mg overnight DST was performed as part of the diagnostic evaluation in selected women with hirsutism, and post-DST DHEA-S levels were assessed in those with elevated baseline levels. This approach allowed us to evaluate DHEA-S changes after short-term dexamethasone exposure without requiring a multi-day protocol. However, adequate cortisol suppression does not necessarily validate the assessment of DHEA-S dynamics. Because DHEA-S has a longer half-life and distinct metabolic characteristics compared to cortisol, its response to short-term dexamethasone may be delayed or incomplete. Therefore, DHEA-S non-suppression after a single overnight DST should be interpreted with caution, as it may reflect incomplete suppression, interindividual variability in adrenal responsiveness, or a functional adrenal contribution, rather than definitive evidence of adrenal autonomy.

DHEA-S is produced almost exclusively by the adrenal glands and serves as a reliable marker of adrenal androgen production (^2^,^4^). In a series of 12 patients with adrenal carcinoma, only three had normal DHEA-S concentrations, while the remainder showed elevated levels (^7^). This observation supports that in some adrenal carcinomas lacking sulfotransferase activity, DHEA-S levels may remain normal. Thus, while normal DHEA-S values do not exclude adrenal disease, elevated levels are highly suggestive of adrenal-origin androgen excess (^7^,^8^). DHEA-S levels exceeding 700 µg/dL in premenopausal women should raise suspicion for an adrenal source, particularly adrenal neoplasms (^1^,^5^,^11^). This threshold aligns with existing recommendations to perform adrenal imaging in such patients to rule out adrenal tumors, which was performed in our cohort, revealing no adrenal masses. In the high-DHEA-S subgroup, six of 24 (25.0%) achieved normalization, and an additional three (12.5%) achieved ≥50% reduction after DST. The mean percent reduction following 1 mg DST was 43.39%. Therefore, while our findings suggest the 1 mg DST could help identify adrenal autonomy, more studies are needed to determine its utility in the differential diagnosis of adrenal carcinoma.

The non-suppressed group represents a distinct biochemical phenotype. Unsurprisingly, these patients not only had higher baseline and post-DST DHEA-S levels, but also significantly higher total testosterone and 17-OHP levels. The observed association between DHEA-S non-suppression and elevated total testosterone is noteworthy. Elevations in both DHEA-S (adrenal) and 17-OHP (adrenal) alongside total testosterone (adrenal/ovarian), may suggest mixed-source hyperandrogenism underpinned by possible adrenal contribution. Our findings support the observation by Wu and cols. (^12^) that hirsutism frequently has a “mixed” etiology. The non-suppressed group, which exhibited both an adrenal component (elevated DHEA-S) and a probable ovarian component (indicated by both higher mean total testosterone levels and a greater frequency of patients with testosterone >ULN), likely represents this mixed cohort. Nonetheless, the % excess of baseline DHEA-S showed weak but statistically significant positive associations with total testosterone and 17-OHP levels. Although these findings may contribute to the biochemical characterization of this subgroup, they are not sufficient to precisely determine the source of hyperandrogenism or to establish a causal relationship. A notable finding is that the non-suppressed group demonstrated significantly higher total testosterone without a corresponding increase in LH, suggesting an LH-independent mechanism of hyperandrogenism. We propose that this may be driven by potential pathways consistent with the possible adrenal contribution in this group (^11^–^15^).

Elevated testosterone may partly reflect peripheral conversion of adrenal precursors (i.e., DHEA-S and 17-OHP), both significantly elevated in our cohort. This is a key finding, as it suggests that the adrenal contribution in our cohort may not be primarily driven by an exaggerated hypothalamic-pituitary-ovarian axis, but rather by an intrinsic adrenal mechanism, potentially a form of functional adrenal hyperandrogenism (FAH) commonly seen within the wide spectrum of PMOS (^14^,^16^). Previous studies have reported that women with PMOS and elevated DHEA-S (FAH phenotype) tend to be leaner and may exhibit a more favorable metabolic profile with lower insulin resistance compared to other PMOS phenotypes (^15^,^17^,^18^). Although we lacked body mass index (BMI) data and observed no difference in HOMA-IR between groups, this likely reflects the limitations of HOMA-IR as a surrogate marker rather than absence of metabolic alterations.

In our study, 79% of patients were diagnosed with PMOS and only two with LOCAH, indicating that DHEA-S non-suppression predominantly reflects a phenotype within the PMOS spectrum, often referred to as FAH rather than a distinct adrenal disorder. From a clinical standpoint, our ROC analysis showed that % excess of baseline DHEA-S was a strong predictor of DHEA-S suppression. In addition, the optimal cut-off of 32.22% above the age-specific ULN could help clinicians identify the degree of adrenal autonomy. Logistic regression further confirmed this dose-dependent association, showing that greater % excess of baseline DHEA-S was inversely associated with suppression. This finding was further supported by the analysis of the >700 µg/dL threshold. Patients with baseline DHEA-S levels above 700 µg/dL were 14.5 times more likely to exhibit non-suppression. Together, these results provide robust evidence that marked DHEA-S elevation predicts adrenal autonomy.

The strengths of this study include its large sample size, well-defined cohort of hirsute women with elevated DHEA-S, standardized laboratory protocols, and comprehensive hormonal assessments enabling exclusion of other endocrinopathies. However, several limitations must be acknowledged. First, as a retrospective study, detailed clinical characteristics such as hirsutism severity, acne, alopecia, and metabolic parameters (e.g., BMI and lipid profile) were unavailable for all patients, limiting a comprehensive clinical and metabolic characterization. Second, while we excluded CS and overt adrenal tumors, we did not employ gold standard procedures such as adrenal venous sampling to confirm the source of androgen excess, as this is an invasive procedure and therefore not feasible. Third, adrenal magnetic resonance imaging was only performed on a small subset of patients (n = 20) with the highest DHEA-S levels, so small, non-secreting micronodules in other patients cannot be definitively ruled out. Fourth, PMOS diagnoses were retrieved from patients’ medical records; hence, ultrasonographic assessments were not standardized across a single radiologist. Finally, serum dexamethasone and ACTH levels were not measured post-DST; nevertheless, all patients in the final analysis demonstrated adequate cortisol suppression after 1-mg overnight DST test, supporting sufficient dexamethasone effect and appropriate test performance.

In conclusion, the 1-mg overnight DST may serve as a practical tool to assess DHEA-S suppressibility in hyperandrogenic women, complementing its established role in cortisol evaluation. Lack of DHEA-S suppression was associated with greater baseline DHEA-S elevation. In our cohort, both the % excess above the age-specific ULN (cut-off 32.22%) and an absolute DHEA-S level >700 µg/dL were useful in identifying a subgroup with the non-suppressed 1-mg DST phenotype. This phenotype was associated with higher total testosterone and 17-OHP levels despite similar LH, suggesting a possible functional adrenal contribution within the broader PMOS-related hyperandrogenism spectrum. Prospective studies are warranted to externally validate these cut-offs and clarify long-term metabolic and reproductive outcomes for this subgroup.

## Data Availability

datasets related to this article will be avail­able upon request to the corresponding author.
